# HPA AXIS RELATED GENES AND RESPONSE TO PSYCHOLOGICAL THERAPIES: GENETICS AND EPIGENETICS

**DOI:** 10.1002/da.22430

**Published:** 2015-10-07

**Authors:** Susanna Roberts, Robert Keers, Kathryn J Lester, Jonathan R. I. Coleman, Gerome Breen, Kristian Arendt, Judith Blatter‐Meunier, Peter Cooper, Cathy Creswell, Krister Fjermestad, Odd E. Havik, Chantal Herren, Sanne M. Hogendoorn, Jennifer L. Hudson, Karen Krause, Heidi J. Lyneham, Talia Morris, Maaike Nauta, Ronald M. Rapee, Yasmin Rey, Silvia Schneider, Sophie C. Schneider, Wendy K. Silverman, Mikael Thastum, Kerstin Thirlwall, Polly Waite, Thalia C. Eley, Chloe C. Y. Wong

**Affiliations:** ^1^ MRC SocialGenetic and Developmental Psychiatry (SGDP) CentreInstitute of PsychiatryPsychology and NeuroscienceKing's College LondonLondonUnited Kingdom; ^2^ School of PsychologyUniversity of SussexUnited Kingdom; ^3^ National Institute for Health Research Biomedical Research CentreSouth London and Maudsley National Health Service TrustUnited Kingdom; ^4^ Department of Psychology and Behavioural SciencesAarhus UniversityAarhusDenmark; ^5^ Department of PsychologyUniversity of BaselBaselSwitzerland; ^6^ School of Psychology and Clinical Language SciencesUniversity of ReadingUnited Kingdom; ^7^ Department of PsychologyStellenbosch UniversitySouth Africa; ^8^ Anxiety Disorders Research NetworkHaukeland University HospitalBergenNorway; ^9^ Department of Forensic PsychiatryUniversity of Basel Psychiatric ClinicsBaselSwitzerland; ^10^ Department of Child and Adolescent Psychiatry/De BasculeAcademic Medical CentreAmsterdamThe Netherlands; ^11^ Centre for Emotional HealthDepartment of PsychologyMacquarie UniversitySydneyAustralia; ^12^ Department of PsychologyRuhr‐Universität BochumBochumGermany; ^13^ Department of Clinical Psychology and Experimental PsychopathologyUniversity of GroningenThe Netherlands; ^14^ Child Anxiety and Phobia ProgramDepartment of PsychologyFlorida International UniversityMiamiUSA; ^15^ Child Study CenterYale University School of MedicineNew HavenConnecticutUSA

**Keywords:** anxiety, child/adolescent, cognitive behavior therapy, genetics, treatment, biological markers, therapygenetics, DNA methylation, HPA axis, FKBP5, glucocorticoid receptor

## Abstract

**Background:**

Hypothalamic–pituitary–adrenal (HPA) axis functioning has been implicated in the development of stress‐related psychiatric diagnoses and response to adverse life experiences. This study aimed to investigate the association between genetic and epigenetics in HPA axis and response to cognitive behavior therapy (CBT).

**Methods:**

Children with anxiety disorders were recruited into the Genes for Treatment project (GxT, *N* = 1,152). Polymorphisms of *FKBP5* and *GR* were analyzed for association with response to CBT. Percentage DNA methylation at the *FKBP5* and *GR* promoter regions was measured before and after CBT in a subset (*n* = 98). Linear mixed effect models were used to investigate the relationship between genotype, DNA methylation, and change in primary anxiety disorder severity (treatment response).

**Results:**

Treatment response was not associated with *FKBP5* and *GR* polymorphisms, or pretreatment percentage DNA methylation. However, change in *FKBP5* DNA methylation was nominally significantly associated with treatment response. Participants who demonstrated the greatest reduction in severity decreased in percentage DNA methylation during treatment, whereas those with little/no reduction in severity increased in percentage DNA methylation. This effect was driven by those with one or more *FKBP5* risk alleles, with no association seen in those with no *FKBP5* risk alleles. No significant association was found between *GR* methylation and response.

**Conclusions:**

Allele‐specific change in *FKBP5* methylation was associated with treatment response. This is the largest study to date investigating the role of HPA axis related genes in response to a psychological therapy. Furthermore, this is the first study to demonstrate that DNA methylation changes may be associated with response to psychological therapies in a genotype‐dependent manner.

## INTRODUCTION

Hypothalamic–pituitary–adrenal (HPA) axis functioning is known to play an important role in reactivity to stress, and impairments in this system have been widely implicated in psychiatric disorders such as anxiety.^1^, ^2^, ^3^ Stress exposure rapidly stimulates glucocorticoid secretion, activating the “fight or flight” response. Termination of this response involves binding of glucocorticoids (i.e., cortisol) at the glucocorticoid receptor. FK506‐binding protein 51(*FKBP5*) acts as a functional negative regulator of glucocorticoid receptor sensitivity by reducing binding affinity^4^ and restricting nuclear translocation.^5^ *FKBP5* mRNA expression has been shown to be regulated by glucocorticoids via glucocorticoid response elements, creating an ultrashort negative feedback loop. Impaired negative feedback in this process can lead to prolonged or excessive stress responses, as seen in anxiety disorders.^1^

Genetic variation of *FKBP5* has been found to interact with early trauma or childhood adversity to predict negative outcomes later in life, such as susceptibility to posttraumatic stress disorder (PTSD), depression, and suicidality.^6^, ^7^, ^8^, ^9^, ^10^ Recent molecular research has suggested that genotype‐dependent structural differences in *FKBP5* may give rise to differential epigenetic changes following early‐life stress^11^ and subsequently functional alterations in the responsiveness of *FKBP5* to glucocorticoid receptor activation.^11^ Epigenetic modifications of the glucocorticoid receptor gene (*NR3C1*, referred to here as *GR*) have also been associated with early‐life experiences in both rats and humans. Studies of early experiences in rats found that DNA methylation of the *GR* promoter region was altered by maternal care, which in turn was associated with *GR* expression and HPA responses to stress.^12^ In humans, stressful life events (e.g., trauma and abuse) have been associated with higher DNA methylation at the *GR* promoter region^13^, ^14^, ^15^, ^16^, ^17^, ^18^ as well as differential GR expression and biological markers of HPA‐axis activity, such as salivary cortisol.^13^, ^14^ Furthermore, *GR* methylation has been implicated in the development of PTSD following trauma.^19^ Together, these findings suggest that *FKBP5* and *GR* genetic and epigenetic differences may influence responsiveness to stressful environments, and the development of stress‐related psychiatric disorders.

There is also increasing evidence that some genetic factors thought to reflect vulnerability to stressful environments may also be associated with more positive outcomes in low‐stress or enriching environments.^20^ Few tests of this differential susceptibility hypothesis have focused on genes involved in stress reactivity, though recent research suggests that the risk allele of *FKBP5* SNP *rs1360780* may be associated with increased risk of depressive symptoms in victimized girls, and also lower depressive symptoms in those with low levels of peer victimization.^21^ Given the role of HPA‐axis genes such as *FKBP5* and *GR* in the biological response to environmental stimuli, they are plausible candidates for investigation of response not only limited to adverse life experiences such as trauma and abuse, but also to positive experiences such as psychological therapies.

Anxiety disorders are associated with high levels of stress. They can be highly debilitating, have the poorest prognosis when they begin early, and show high continuity into adulthood.^22^, ^23^ Psychological therapies such as cognitive behavior therapy (CBT) represent an effective treatment option for many children with anxiety disorders, with ∼60% of children remitting following CBT.^24^ The field of “therapygenetics” is a growing area of research focusing on genetic predictors of outcome in response to psychological therapies.^25^ A small number of candidate genes have been implicated, focusing primarily on polymorphisms and epigenetic regulation of genes involved in brain‐related markers, such as the serotonin transporter gene (*SERT* 26, 27, 28), brain‐derived neurotrophic factor (*BDNF* 29), and nerve growth factor (*NGF* 30), among others.^31^ One study has examined *FKBP5* genotype with respect to therapy outcome, finding an association between the SNP *rs1360780* and the effectiveness of narrative exposure therapy for PTSD (*N* = 43^32^). With respect to DNA methylation, a study of PTSD in veterans indicated that DNA methylation of *FKBP5* and *GR* may be associated with response to exposure‐based psychotherapy in PTSD (*N* = 16^33^). Pretreatment *GR* methylation predicted treatment response, whereas a decrease in *FKBP5* methylation across treatment was associated with recovery. To date, no studies have combined genetic and epigenetic data when looking at the association between HPA axis related genes and response to CBT.

In this study, we tested the association between polymorphisms of *FKBP5* and *GR* and response to CBT in children with anxiety disorders (*N* = 1,152), and examined change in DNA methylation at specific regions of these genes during the course of CBT in a subset of the sample (*n* = 98). Our first hypothesis was that there would be a significant effect of stress reactivity related genotypes on change in anxiety severity. Our second hypothesis was that there would be an association between *FKBP5* and *GR* methylation and response to CBT, looking at prediction by pretreatment DNA methylation, and change in DNA methylation during treatment. Previous research has demonstrated that DNA methylation patterns are linked to DNA sequence variation at a large number of locations across the genome,^34^, ^35^ and allele‐specific DNA methylation has also been reported at in *FKBP5*.^11^ Therefore, our third hypothesis was that there would be an interaction between *FKBP5* and *GR* genotypes and DNA methylation on CBT response. This is the largest study to date that has investigated the association of *FKBP5* and *GR* with response to a psychological therapy, and the first study of DNA methylation and response to a psychological therapy to include genotypic information.

## PARTICIPANTS

### STUDY OVERVIEW

All participants come from the Genes for Treatment (GxT) Study, an international multisite collaboration designed to identify predictors of outcome following CBT for child anxiety.^28^, ^36^

### PARTICIPANTS AND TREATMENT

Subjects aged 5–18 (mean: 9.8 years) met DSM‐IV criteria for primary diagnosis of an anxiety disorder. Exclusion criteria included significant physical/intellectual impairment, psychoses, and concurrent treatment. All participants (*N* = 1,152) completed a full course of CBT either as part of a trial or treatment as usual at one of 11 sites. Detailed sample characteristics and site‐specific trial details can be found in the Supporting Information and in Table 1.

**Table 1:** Sample demographics and clinical information

			Sydney		Reading and Oxford							
Characteristic	Site	Whole sample	All	Subgroup	All	Subgroup	Aarhus	Bergen	Bochum	Groningen	Florida	Basel
Sample (*N*)		1152	617	74	217& 3	23& 1	121	110	42	34	5	3
Age: mean (SD)		9.9(2.2)	9.4(1.9)	9.3(1.9)	9.6(1.8)	9.4(1.4)	11.1(2.4)	11.3(2.1)	10.6(1.9)	12.1(3.1)	11.0(2.4)	8.7(2.3)
Gender: female (%)		50.7	48.9	48.6	52.3	58.3	57.9	53.7	50	47.1	40	0
Primary diagnosis	Pretreatment	6.3(1.0)	6.4(0.9)	6.5(0.8)	5.7(0.8)	5.5(1.1)	6.6(1.2)	6.9(1.2)	6.7(1.1)	6.2(1.0)	6.0(1.0)	6.7(0.6)
Severity: mean (SD)	Posttreatment	3.2(2.2)	3.3(1.8)	3.3(1.7)	2.7(2.4)	3.4(2.8)	2.9(2.6)	4.8(2.3)	2.1(2.3)	2.6(1.4)	3.0(2.6)	4.3(2.1)
	Follow‐up	2.5(2.2)	2.8(1.8)	2.6(1.7)	1.8(2.3)	1.9(2.7)	2.0(2.5)	3.8(2.6)	1.7(0.9)	0.6(1.6)	0.0(0.0)	4.7(1.2)
Ethnicity: white‐European ancestry; %known (%total)		87.0(64.3)	82.6(61.8)	81.5(71.6)	88.2(67.7)	77.8(58.3)	97.4(92.6)	97.0 (29.1)	91.7(78.6)	96.9(91.2)	0.0(0.0)	100.0(100.0)

### CLINICAL MEASURES

Anxiety disorders were assessed before and after treatment, and at follow‐up (3, 6, or 12 months after conclusion of treatment). Diagnoses were made with the Anxiety Disorders Interview Schedule for DSM‐IV (ADIS‐IV‐C/P; 37) at all sites except for Bochum and Basel, where the German equivalent (Kinder‐DIPs) was used.^38^ Clinical severity ratings (CSRs) were usually based on composite parent and child reports, and were assigned on a scale of 0–8.^36^ Treatment response was defined as the change in primary anxiety disorder severity from pretreatment to follow‐up. A diagnosis was made when the child met diagnostic criteria and received a CSR of 4 or more. Remission was regarded as the absence of the primary anxiety according to diagnostic criteria, as determined by the clinicians at the follow‐up interview. Details of primary diagnoses included can be found in the Supporting Information.

## METHODS

### GENOTYPING

Participants provided DNA using buccal swabs or Oragene saliva samples (DNA Genotek, Ottawa, Canada). DNA was extracted using established procedures.^39^, ^40^ Genotypes for five FKBP5 polymorphisms (*rs1360780, rs3800373, rs9296158, rs9470080, rs4713916*) and five GR polymorphisms *(rs6189, rs6190/R23K, rs6195/N363S, rs6198/GR‐9beta, rs41423247/BcII1*) were genotyped by LGC Genomics (Hoddesdon, UK) using validated arrays with KASP technology or were obtained from the Illumina Core Exome array. SNPs were included if they could be imputed with >90% completeness, and an information score of >.8. Four additional markers were genotyped using both platforms to check for agreement across these methodologies, and showed an average of 98% consensus on genotype calls. Using these cutoffs, data were available for all *FKBP5* SNPs, and two *GR* SNPs (*rs6195/N363S* and *rs4142324/BcII1*).

### DNA METHYLATION

A subset of participants had DNA samples available for two time points—before and after treatment. All samples included in DNA methylation analyses were derived from buccal swabs. These participants were treated at Sydney, Australia (*n* = 74) and Reading or Oxford, UK (*n* = 24). In these samples, extracted genomic DNA (510 ng) was treated with sodium bisulfite using the EZ‐96 DNA Methylation Kit (Zymo Research, USA). Sequences were chosen based on their proximity to the gene promotor region and CpG islands—further rationale details are provided in the Supporting Information. Bisulfite‐PCR primers for the *FKBP5* and *GR* amplicons were designed using the Sequenom EpiDesigner software (Supporting Information Table S1). PCR amplification was conducted using 40 cycles at an annealing temperature of 63/56°C (*FKBP5/GR*), with Qiagen HotStarTaq DNA Polymerase and Sequenom MassCLEAVE tagged primers. Percentage DNA methylation was quantitatively measured using the Sequenom EpiTYPER system (Sequenom, San Diego, CA, USA). Artificially fully methylated and unmethylated samples were included for quality control, and any probes detecting an average DNA methylation of <5% were dropped from further analyses. Following stringent quality control, quantitative methylation data were available for four *FKBP5* CpG probes and four *GR* CpG probes (*n* = 98), referred to as CpG 1–4 in the results.

### ETHICS APPROVAL

Research was conducted in compliance with the standards outlined by the Declaration of Helsinki. All trials and collection of samples were approved by site‐specific Human Ethics and Biosafety Committees. Parents provided informed consent, children assent. The storage and analysis of DNA was approved by the King's College London Psychiatry, Nursing and Midwifery Research Ethics Sub‐Committee.

## ANALYSES

### TREATMENT OUTCOME

To maximize power, we focused our analyses on *change in primary anxiety disorder severity* from *pretreatment* to *follow‐up*. We refer to this from hereon as *treatment response*. Significant predictors were then considered with respect to *remission* of the primary diagnosis. Follow‐up assessments were usually conducted 6 months after the conclusion of treatment (participants with 3‐ and 12‐month follow‐ups were also included; see Supporting Information).

### ETHNICITY

Self‐reported ethnicity was available for 73.9% of the sample, of whom 87.0% were of white‐European ancestry. Ethnicity was unrelated to genotypic proportions (all SNPs *P* >.05, see Supporting Information Table S2) or treatment outcome (β = .01, *P* =.276).

#### Genotype

High‐quality genotype and follow‐up outcome data were available for 924 children for *FKBP5* markers, and 1,000 children for *GR*.

Linear mixed effect models were used to test the effect of *FKBP5* and *GR* polymorphisms on treatment response. Treatment trial was included as a higher order random effect in order to account for differences between trials (see Supporting Information and Table 1). Gender, age, and baseline severity were included as covariates, with age and baseline severity centered at the mean. Analyses also included the linear and quadratic effects of time as covariates. As all SNPs showed a low frequency of the homozygous risk allele genotype, polymorphisms were coded to reflect a dominant model, with homozygotes of the “non‐risk” allele versus “risk” allele carriers. This is in accordance with previous studies of response to environmental stimuli and psychiatric outcomes 32, 41 and interactions between genotype and DNA methylation.^11^

#### DNA Methylation

Prediction of treatment response was assessed using pretreatment percentage DNA methylation values. Change in DNA methylation from pre‐ to posttreatment was also calculated for all CpG sites. Linear mixed effect analyses were used to test the association between percentage DNA methylation (both pretreatment and change) and change in clinical severity from pretreatment to follow‐up. Age (centered), gender, and baseline severity (centered) were included as covariates in all models, and treatment trial was included as a higher order random effect.

#### Genotype and DNA Methylation Interaction Analyses

To test the effect of genotype on the association between percentage DNA methylation and treatment outcome, linear mixed effect models were run as previously described, including an interaction term (genotype × DNA methylation). As before, prediction of treatment response was assessed using pretreatment percentage DNA methylation. Change in percentage DNA methylation from pre‐ to posttreatment was also tested for association with treatment response.

*FKBP5*. A high correlation was observed between all *FKBP5* SNPs (*r* = .72–.99), and all SNPs fit the same pattern of genotypic distribution as *rs1360780*. Previous research has indicated that *rs1360780* may play a functional role in the interaction between environmental stimuli and DNA methylation,^11^ with genotype coded using a dominant model. Therefore, *FKBP5* genotype was collapsed to reflect those with no “risk” alleles, versus those with one or more risk allele (0 = 41.3%, 1–5 = 58.7%^1^ ).

*GR*. Only one *GR* SNP (*rs4142324*) showed sufficient variability in the available sample to be included in the interaction analyses. As before, a dominant model (GC/CC vs. GG) was used for *GR* genotype.

All analyses were performed in STATA version 11.2 .^42^

#### Multiple Testing

To correct for multiple testing, revised significance thresholds were estimated. These values were calculated using Matrix Spectral Decomposition (MatSpD)^45^ to determine the number of independent variables in the data. First, for the genotypic analyses MatSpD indicated that the seven SNPs tested in the genotypic analyses corresponded with 4.8 independent variables (α <.0104). Second, for analyses of DNA methylation the eight CpG sites examined corresponded with 7.8 independent variables (α <.0064). Third, to account for the additional effect of genotype included in the interaction analyses (two possibilities; risk vs. no‐risk), this value was divided by two, giving a threshold of α <.0032.

## RESULTS

Clinical outcomes were comparable to previously reported estimates. Following treatment, 54.4/38.3% of the sample was free of their primary/all anxiety diagnoses, respectively. By follow‐up, these rates rose to 65.6% for the primary diagnosis, and 49.0% for all anxiety diagnoses. Mean severity at pretreatment was 6.3 (sd = 1.0), dropping to 3.2 (sd = 2.2) at posttreatment, and 2.5 (sd = 2.2) at follow‐up.

### GENOTYPE AND THERAPY OUTCOMES

There was no significant effect of *FKBP5* or *GR* genotype on treatment response (Table 2).

**Table 2:** Genotype and primary anxiety response

Gene	Polymorphism	Mean primary anxiety response (SE)	β	95% CI	*P*
FKBP5	*rs1360780*	CC = −3.79 (.11) CT/TT = −3.7 (.10)	.01	−.05 to .06	.847
		Age	.00	−.01 to .01	.965
		Sex	.04	−.01 to .09	.150
		Baseline severity	.25	.23 to .28	<.001
		Time	−1.13	−1.17 to 1.08	<.001
		Time2	.18	.17 to .19	<.001
	*rs3800373*	TT = −3.74 (.11) TG/GG = −3.76 (.10)	−.01	−.06 to .04	.668
		Age	.00	−.01 to .02	.739
		Sex	.05	−.00 to .10	.072
		Baseline severity	.25	.22 to .28	<.001
		Time	−1.13	−1.17 to 1.08	<.001
		Time2	.18	.17 to .19	<.001
	*rs4713916*	GG = −3.78 (.11) GA/AA = −3.68 (.11)	.00	−.05 to .06	.931
		Age	.00	−.01 to .02	.557
		Sex	.04	−.01 to .10	.148
		Baseline severity	.25	.22 to .28	<.001
		Time	−1.13	−1.18 to 1.08	<.001
		Time2	.19	.17 to .20	<.001
	*rs9296158*	GG = −3.78 (.11) GA/AA = −3.70 (.10)	.01	−.05 to .06	.791
		Age	.00	−.01 to .02	.705
		Sex	.05	−.00 to .10	.071
		Baseline severity	.25	.22 to .28	<.001
		Time	−1.13	−1.17 to 1.08	<.001
		Time2	.18	.17 to .19	<.001
	*rs9470080*	CC = −3.76 (.12) CT/TT = −3.74 (.09)	.00	−.06 to .05	.933
		Age	.00	−.01 to .02	.768
		Sex	.05	−.00 to .10	.064
		Baseline severity	.25	.22 to .28	<.001
		Time	−1.13	−1.17 to 1.08	<.001
		Time2	.18	.17 to .19	<.001
GR	*rs6195*	AA = −3.76 (.07) AG = −3.58 (.27)	−.04	−.15 to .08	.542
		Age	.00	−.01 to .02	.719
		Sex	.05	.00 to .11	.046
		Baseline severity	.25	.22 to .28	<.001
		Time	−1.12	−1.17 to 1.08	<.001
		Time2	.18	.17‐.19	<.001
	*rs41423247*	GG= −3.82 (.09) GC/CC = −3.72 (.12)	.00	−.05 to .06	.959
		Age	.00	−.01 to .01	.850
		Sex	.04	−.01 to .10	.111
		Baseline severity	.24	.22 to .27	<.001
		Time	−1.13	−1.18 to 1.09	<.001
		Time2	.18	.17 to .19	<.001

Primary anxiety response is defined as the change in severity for the primary diagnosis from pretreatment through follow‐up time points.

### DNA METHYLATION AND RESPONSE TO THERAPY

#### Preliminary Analyses

There was no time‐point effect on DNA methylation (*n* = 98), with no significant differences between pre‐ and posttreatment DNA methylation detected for any CpG site in either *FKBP5* or *GR* (Supporting Information Table S3).

#### Prediction

Analyses of pretreatment *FKBP5* and *GR* methylation showed no association with treatment response at any CpG site (Supporting Information Table S4).

#### Change

**Figure 1:**
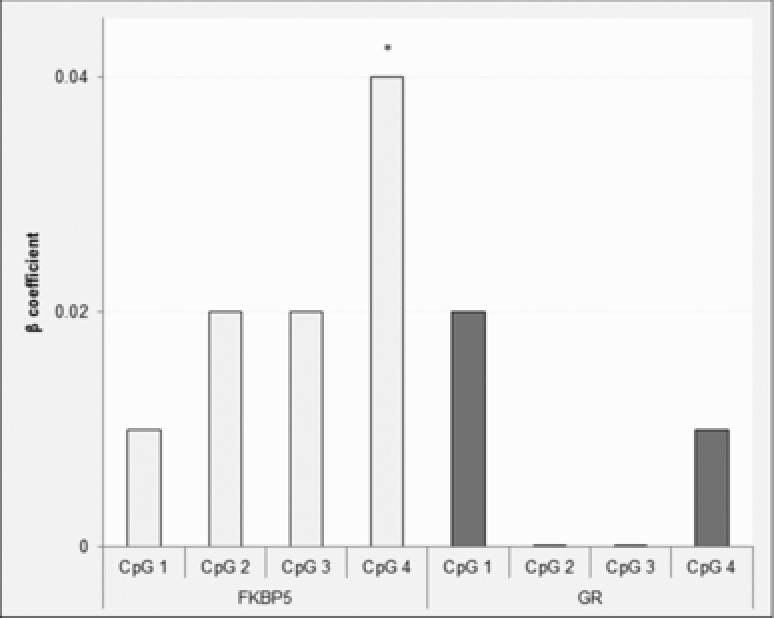
Association between change in *FKBP5* and *GR* methylation and primary anxiety response. *Note*: The values given for the beta (β) coefficient are absolute, and refer to the main effect of change in percentage DNA methylation on primary anxiety response (change in primary anxiety disorder severity from pretreatment to follow‐up). *P = .0069, nominally significant.

*FKBP5*. There was a significant association between change in CpG 4 methylation from pre‐ to posttreatment, and treatment response (β = .04, *P* = .0069, Figure 1). Specifically, increased DNA methylation was associated with a smaller reduction in symptom severity, whereas a decrease in DNA methylation was associated with a greater reduction in symptom severity. However, this effect did not remain significant when corrected for multiple testing (*α < .0064)*. There was no significant association between change in percentage DNA methylation at CpG sites one to three and treatment outcomes (Figure 1).

*GR*. Change in DNA methylation was not significantly associated with treatment outcome for any *GR* CpG site (Figure 1).

### INTERACTION BETWEEN GENOTYPE AND DNA METHYLATION ON TREATMENT OUTCOMES

#### Preliminary Analyses

There was no effect of genotype on pretreatment percentage DNA methylation or change in percentage DNA methylation at any CpG site for *FKBP5* or *GR* (Supporting Information Table S5).

#### Prediction

There was no significant effect of an interaction between genotype and pretreatment percentage DNA methylation on treatment response for either *FKBP5* or *GR* (Supporting Information Table S6).

#### Change

**Figure 2:**
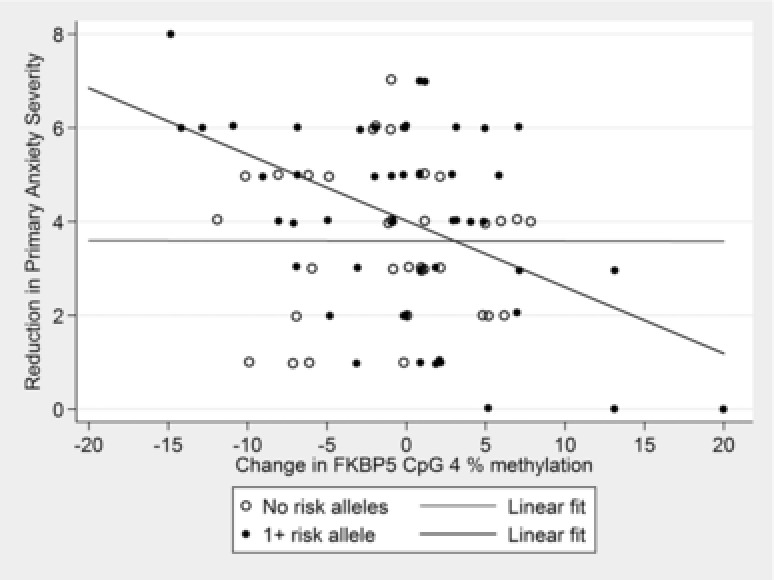
Association between change in *FKBP5* methylation and primary anxiety response as a function of *FKBP5* genotype. *Note*: Treatment response is depicted as reduction in primary anxiety disorder severity, which takes into account the baseline (pretreatment) severity, thus reflecting more accurately the models tested (which included pretreatment severity as a covariate). Note that a larger reduction in symptom severity represents a greater response to treatment. No risk alleles: β = .002, 95% CI = −.04–.05, *P* = .905 n = 34; 1+ risk alleles: β = .06, 95% CI = .03–.10, *P* = 1.3 × 10^−4^, n = 52. Interaction: β = .14, 95% CI = .02–.27, *P* = .028.

**Figure 3:**
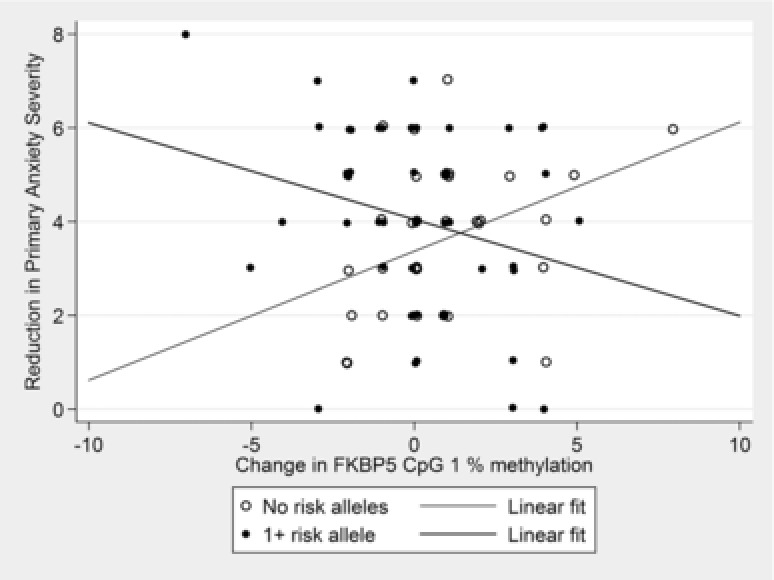
Interaction between change in FKBP5 methylation and FKBP5 genotype on primary anxiety response. *Note*: Treatment response is depicted as reduction in primary anxiety disorder severity, which takes into account the baseline (pretreatment) severity, thus reflecting more accurately the models tested (which included pretreatment severity as a covariate). Note that a larger reduction in symptom severity represents a greater response to treatment. Interaction β = .20, 95% CI = .05–.35, *P* = .008.

*FKBP5*. At CpG 4, there was a nominally significant interaction between change in percentage DNA methylation, *FKBP5* genotype, and treatment response (β = .14, 95% CI =.02–.27, *P* = .028, Figure 2), although this was not significant when correcting for multiple testing. Interestingly, in those with the risk allele genotype, change in CpG 4 percentage DNA methylation was associated with treatment response (β = .06, 95% CI =.03–.10, *P* = 1.3 × 10^−4^), but not in those with no risk alleles (β = .002, 95% CI =−.04 to .05, *P* = .905). The same pattern of effects was observed in post hoc analyses of primary anxiety diagnosis remission (See Supporting Information). The association between change in percentage DNA methylation and treatment response in participants with the risk genotype remained significant when corrected for multiple testing. There was also a nominally significant interaction between change in DNA methylation at CpG 1, *FKBP5* genotype, and treatment response (β = .20, 95% CI =.05–.35, *P* = .008). At this CpG site, those with the risk allele showed the same pattern of DNA methylation change as for CpG 4, while those with no risk alleles showed the opposite pattern of effect (Figure 3). There was no effect of an interaction between genotype and change in percentage DNA methylation for CpG sites 2 and 3.

*GR*. There was no significant effect of an interaction between change in percentage DNA methylation and *GR* genotype on treatment response for any CpG site (Supporting Information Table S7).

## DISCUSSION

This study utilizes a large sample to test the association between HPA‐axis related genes and response to psychological therapy, including both genetic and epigenetic information as predictors of treatment outcome. We found suggestive evidence for an association between change in *FKBP5* methylation and treatment response. This is comparable to previous reports of *FKBP5* methylation change and psychological therapy response.^33^ Of note, in our sample this effect was largely driven by those with the “risk” genotype, suggesting that this association may be genotype dependent. Intriguingly, allele‐specific methylation of *FKBP5* intron 7 was previously demonstrated to be associated with childhood trauma,^11^ which suggests that this may be one mechanism by which negative experiences early in life affect long‐term susceptibility to stress‐related disorders, such as anxiety and CBT. The current study focuses on a different region of the *FKBP5* gene, but our findings indicate that genotype‐dependent change in *FKBP5* methylation may also be associated with positive environmental influences; in this case, psychological therapy. Furthermore, it is possible that the effects described reflect a molecular mechanism by which some people are more or less reactive to their environment. In our sample, individuals with the *FKBP5* “risk” genotype showed greater variability in DNA methylation changes and treatment outcome. A greater response to therapy (i.e., greater reduction in severity) was associated with decreases in *FKBP5* methylation, whereas a poorer response to therapy was associated with increases in *FKBP5* methylation. This is consistent with a differential susceptibility framework, whereby individuals with particular genetic factors may be more vulnerable to their environment, “for better or for worse.”^20^ The same pattern of association was seen in risk genotype individuals for a further CpG site in the same region, while no‐risk genotype individuals showed an opposite pattern of effect. These findings highlight the allele‐specific nature of DNA methylation changes at this region in our sample.

We did not find any effect of *FKBP5* or *GR* genotype, or pretreatment DNA methylation on treatment response. Previous research suggested that the *FBKP5* genotype *rs1360780* is associated with response to narrative exposure therapy in adults with PTSD (*N* = 43).^32^ We did not replicate this finding in the current large sample of response to CBT in children with anxiety disorders (*rs1360780, n* = 912). In a sample of this size (>900), we were well powered to detect even small‐medium effect sizes (e.g., >80% power to detect an effect size of *d* = .26 at a significance level of α = .05). Moreover, we did not observe a significant association between pretreatment or change in *GR* methylation and treatment outcome. This is in contrast to the previously reported finding that pretreatment *GR* methylation predicted treatment response in veterans with PTSD.^33^ Higher *GR* methylation has been associated with early‐life adversity, such as childhood abuse or maltreatment, or prenatal exposure to maternal anxiety, depression, and stress^13^, ^14^, ^15^, ^16^, ^18^ In these studies, higher methylation was associated with reduced GR expression^13^ and also increased salivary cortisol responses to stress.^14^ The absence of an association in our sample may suggest that *GR* methylation levels are more susceptible to traumatic experiences, and may be more stable over time. However, it should also be noted that our DNA methylation analyses relied on a smaller subsample, and would ideally be replicated in a larger dataset.

To date, only a handful of studies have examined DNA methylation patterns with response to psychological therapy. A previous study of PTSD in veterans found that pretreatment *GR* methylation patterns predicted treatment outcome while *FKBP5* methylation decreased across the course of therapy in treatment responders, but increased in nonresponders.^33^ Another study examining response to intensive dialectical behavior therapy in adults with borderline personality disorder demonstrated differential patterns of *BDNF* methylation change associated with scores of depression, hopelessness, and impulsivity following treatment.^29^ Finally, we previously showed that one CpG site upstream of the *SERT* promoter increased in DNA methylation during the course of therapy in treatment responders, but decreased in nonresponders.^27^ Intriguingly, in all of these studies responders and nonresponders showed a difference in the direction of DNA methylation change.

This study is the largest to date to examine genetic markers involved in stress reactivity for association with response to CBT. Furthermore, this is the first study to demonstrate that genotype‐dependent changes in DNA methylation are associated with response to psychological therapy. However, there are some limitations. First, analyses using the whole dataset contain participants with a range of anxiety diagnoses, treated with different CBT modalities, leading to a high level of heterogeneity. Second, while we were able to assess DNA methylation at both pre‐ and posttreatment, we have no DNA samples to measure percentage DNA methylation for these markers at follow‐up. In this study, we find genotype‐dependent changes in methylation during treatment are associated with treatment outcomes at *follow‐up*. Previous studies investigating both genetic^26^, ^30^, ^32^ and epigenetic^26^, ^27^ markers of response to psychological therapy have also demonstrated the largest effects at follow‐up. Future research of predictors and markers of response to psychological therapies would be improved by including this time point. Third, studies investigating biological and molecular changes in living patients require the use of peripheral tissue as a proxy for more specific tissues of interest. Furthermore, the use of a child sample in this study necessitated the use of minimally invasive DNA sampling techniques, namely buccal swabs and saliva. However, while tissue‐specific differences in DNA methylation have been documented,^43^ it has been suggested that buccal swabs may be more representative than other peripheral tissues such as blood in epigenetic studies.^44^ This is due to a reduced cell heterogeneity in buccal swabs, and because they originate from the same developmental pathways as brain tissue.^44^ Fourth, information on exposure to trauma prior to treatment was not available for this sample. Traumatic experiences in childhood have been demonstrated to have lasting effects on DNA methylation of both the *FKBP5* and *GR* genes.^11^, ^12^, ^14^, ^15^, ^16^, ^17^ Therefore, we are unable to make any conclusions regarding other environmental factors that may have influenced baseline DNA methylation and subsequent changes in DNA methylation during active treatment. Finally, we could not make any inferences about the potential functionality of the genotype‐dependent methylation changes observed as we were unable to measure *FKBP5* or *GR* expression in this cohort. Previous studies have demonstrated that *FKBP5* expression is associated with DNA methylation, although it should be noted that this study focused on methylation of *FKBP5* intron 7.^11^ Furthermore, a previous study *of FKBP5* and *GR* and response to psychological therapy found that endocrine markers of HPA‐axis activity were predictive of *FKBP5* and *GR* gene expression at follow‐up, and that plasma cortisol was correlated with expression of both genes.^33^ Future studies would benefit from utilizing an integrative approach, by combining genotype, DNA methylation, and mRNA expression data when examining these markers.

## CONCLUSION

In this study, we provide preliminary evidence that response to a psychological therapy may be associated with genotype‐dependent changes in DNA methylation of *FKBP5*, which is related to HPA‐axis functioning and responsiveness to stress. These results require replication, but add to the growing literature demonstrating that response to environmental influences (both positive and negative) is associated with changes at a biological level.

## Financial support

Combined study supported by UK Medical Research Council grant G0901874/1 to Eley. Individual trials support by Australian Research Council grant DP0878609 to Hudson, Donald, Rapee, and Eley; Australian NHMRC grants to Rapee, Hudson, Lyneham, Mihalopolous (1027556), Lyneham, Hudson, and Rapee (488505), and Hudson and Rapee (382008); TrygFonden grant (7‐10‐1391) to Thastum and Hougaard; Edith og Godtfred Kirk Christiansens Fond grant (21‐5675) to Thastum; Swiss National Science Foundation grant (105314‐116517) to Schneider; Western Norway Regional Health Authority grants to Havik (911253) and Heiervang (911366); NIMH R01 (MH079943) to Silverman; UK NIHR grants to Creswell, Cooper, McIntosh, and Willetts (PB‐PG‐0110‐21190) and Cooper, Creswell, Willetts, and Sheffied (PB‐PG‐0107‐12042); UK Medical Research Council Grants to Cooper and Creswell (09‐800‐17), Thirlwall, Cooper, and Creswell (G0802326), Waite, Creswell, and Cooper (G1002011), and Creswell (G0601874). UK Medical Research Council Grants also supported Keers (MR/K021281/1) and Lester (MR/J011762/1), and Lester received a Jacobs Foundation Young Investigator Award. Grant 09‐800‐17 was managed by NIHR on behalf of the MRC‐NIHR partnership. This study presents independent research partly funded by the National Institute for Health Research (NIHR) Biomedical Research Centre at South London and Maudsley NHS Foundation Trust and King's College London. The views expressed are those of the author(s) and not necessarily those of the NHS, the NIHR, or the Department of Health.

## Conflict of interest

Rapee, Hudson, and Lyneham are authors of the Cool Kids program, but receive no direct payments. Creswell was joint author of book used in treatment within the overcoming trial and receives royalties from sales of the book. Schneider is an author of the Diagnostisches Interview bei psychischen Störungen im Kindes‐ und Jugendalter, for which she receives royalties. Silverman is an author of the Anxiety Disorders Interview Schedule for Children, for which she receives royalties. Breen is a consultant in Preclinical Genomics for Eli Lilly, for which he receives personal fees, and also has grant funding from Eli Lilly. All other authors declare no financial interests.

## Supporting information

Disclaimer: Supplementary materials have been peer‐reviewed but not copyedited.

Supporting Material

## Footnotes

1 Note: Only 11.7% of the sample had one to four risk alleles, therefore the majority of those in the “1+ risk alleles” group had five risk alleles.
