# Supplementary material for: HPA AXIS RELATED GENES AND RESPONSE TO PSYCHOLOGICAL THERAPIES: GENETICS AND EPIGENETICS
Source: Depress Anxiety. 2015 Oct 7;32(12):861–70. doi: 10.1002/da.22430 (PMC4982063; doi:10.1002/da.22430)
Supplement: Supplementary file 1 — Supporting Material [file DA-32-861-s001.docx]

**Supplementary information:**

**Sample characteristics and site specific trial details**

Unless otherwise specified, clinical trials included all primary anxiety disorder diagnoses. All sites made secondary anxiety disorder diagnoses where appropriate.

**Sydney, Australia (n =** 617**).** Participants aged 6-18 were recruited from the Centre for Emotional Health, Macquarie University, Sydney. All participants completed the Cool Kids program [^1^](#_ENREF_1), with 10-12 family sessions involving the parents (the majority of which were conducted in groups; 8% of the sample’s DNA were collected retrospectively). Variations on this treatment program include a subgroup from previous randomised trials who received group, individual or phone-based CBT sessions [^2^](#_ENREF_2); participants from a guided self-help trial with phone support for children in rural Australia [^3^](#_ENREF_3); a group from a trial with additional parental anxiety management [^4^](#_ENREF_4); and those recruited from an ongoing randomised trial of progressive allocation to treatment (Stepped Care).

**Reading and Oxford (n =** 217 & 3**), UK** Participants aged 5-18 were recruited jointly from Reading and Oxford from eight trials at the Berkshire Child Anxiety Clinic (University of Reading) and the Oxfordshire Primary Child and Adolescent Mental Health Service. Participants received treatment in three main themes; one focussing on children with anxious mothers; a set of trials using a parent-guided self-help CBT program; and an online CBT program for adolescents.

*The Mother and Child (MaCh) project* (Creswell et al., In press) Children whose mother also had a current anxiety disorder completed an 8 session manual-based CBT treatment based on the Cool Kids program. The mothers of these children also received extra sessions focussing on their own anxiety and on mother-child interactions.

*Overcoming.* Children were treated with a parent-guided self-help CBT program, comprised of the same primary components as the Cool Kids program [^5^](#_ENREF_5). This consisted of 2-4 in-person sessions and 2-4 telephone sessions. A sub-set of this group with a primary anxiety disorder diagnosis of Social Phobia also received targeted Cognitive Bias Modification Training (CBM-I, [Vassilopoulos et al. ^[6^](#_ENREF_6), Orchard et al., In submission). Additionally, participants with highly anxious parents (screened using DASS or by meeting ADIS criteria) were randomised to groups in a trial including additional sessions for the parents which focussed on strategies for tolerating children’s negative emotions (Hiller et al., In submission). In Oxford, treatment was based on the same basic program, and delivered by primary health workers as part of a feasibility trial [^7^](#_ENREF_7).

*BRAVE*. The final treatment group completed a therapist-supported online CBT program for adolescents (BRAVE, [Spence et al. ^[8^](#_ENREF_8)), consisting of 10 sessions, half with 5 additional parent sessions and half without parent sessions.

**Aarhus, Denmark (n =** 121**).** Participants aged 7-17 years were recruited from the Department of Psychology and Behavioural Sciences, Aarhus University, and all anxiety disorder diagnoses were included. Participants received CBT using the Cool Kids manual (including the adolescent version where appropriate [^9^](#_ENREF_9)). Participants came from two groups; one aged 7-17, from a trial including treatment and waitlist conditions; and another group aged 7-12 from a trial comparing efficacy of traditional group-based treatment with Cool Kids versus a guided self-help version with clinician support (bibliotherapy). In both trials only participants that received in-person CBT were included.

**Bergen, Norway (n =** 110**).** Participants aged 5-13 were recruited from the child part of the “Assessment and Treatment – Anxiety in Children and Adults” study, Haukeland University Hospital, Bergen. Patients referred to outpatient mental health clinics in Western Norway, with a primary diagnosis of separation anxiety, social phobia, or generalized anxiety, received group or individual treatment with the FRIENDS program (4^th^ edition [^10^](#_ENREF_10)^,^[^11^](#_ENREF_11)) in a randomised control trial comparing active treatment with a waitlist condition [^12^](#_ENREF_12).

**Bochum, Germany (n =** 42**).** Participants aged 5-18 were recruited from the Research and Treatment Centre for Mental Health, Ruhr-Universität Bochum. Participants received either exposure-based CBT (8-25 sessions, with sessions occurring at least every 2 weeks), the Coping Cat program [^13^](#_ENREF_13), or a family-based version of CBT specifically designed to target separation anxiety disorder (TAFF [^14^](#_ENREF_14)^,^[^15^](#_ENREF_15)). Diagnoses were provided separately for parent- and child-report. The primary diagnosis was selected as being the most severe from either reporter. If the most severe disorder reported by each was of equal severity but was a different diagnosis, the parent-reported diagnosis was selected.

**Groningen, the Netherlands (n =** 34**).**  Participants aged 8 to 17 were recruited from the Department of Child and Adolescent Psychiatry, University of Groningen. All participants were treated within a randomised control trial of Coping Cat (Dutch version [^16^](#_ENREF_16) including 12 individual child sessions and 2 parent sessions.

**Florida, USA (n =** 5**).** Participants aged 7 to 16 (including all primary anxiety disorder diagnoses except PTSD) were recruited from the Child Anxiety and Phobia Program, Florida International University, Miami. All participants received 12 to 14 hour-long sessions of individual manualised CBT. Additionally, two conditions included parental involvement focussing on different parent skills (Relationship Skills Training or Reinforcement Skills Training).

**Basel, Switzerland (n = 3).** Participants aged 5-13 (all with a primary diagnosis of Separation Anxiety Disorder) were recruited from the Faculty of Psychology, University of Basel. All participants took part in a randomised control trial comparing a family-based version of CBT specifically designed to target separation anxiety disorder (TAFF[^14^](#_ENREF_14)^,^[^15^](#_ENREF_15)) with Coping Cat [^13^](#_ENREF_13). All participants received 16 sessions over 12 weeks.

All treatments were manualised and treatment protocols across sites were comparable for core elements of CBT, including teaching of coping skills, cognitive restructuring and exposure. Treatment modalities fell into three broad groups – individual CBT (25.4%), group based CBT (61.3%) and parent-supported guided self-help (13.3%).

Primary diagnoses included Generalised Anxiety Disorder (GAD; 38.2%), Separation Anxiety Disorder (SAD; 19.9%), Social Anxiety Disorder (23.7%), Specific Phobias (11.2%), or Panic Disorder, Obsessive Compulsive Disorder, Post-Traumatic Stress Disorder, Selective Mutism or Anxiety Disorders Not Otherwise Specified (other anxiety disorders; 7.0%). In the cases with primary selective mutism, a diagnosis of severe social phobia was also given. The selective mutism was considered by the clinician to be primary, the most interfering.

Treatment trials showed significant differences for age (*F*(19, 1151)= 14.77, *p*<0.001) and baseline severity (*F*(19, 1151)= 10.99, *p*<0.001). Treatment trial group was also a significant predictor of treatment response (β = 0.01, *p*= 0.01), and so was included as a higher order random effect in all models to account for differences between trials. Gender proportions did not significantly differ by trial (Χ^2^ = 20.12, *p*=0.387). Genotypic distribution also did not differ by trial (all *p*>0.05).

**Follow-up time-points**

For genotypic analyses, 1152 children had sufficient data to be included in at least one analysis. Treatment outcome was defined as change in primary anxiety disorder severity from pre-treatment to follow-up. The majority of the sample had follow-up at 6 months (66.0%). A smaller number had a follow-up appointment at either 3 months (17.9%) or 12 months (30.0%). Some individuals had follow-up appointments at both 6 and 12 months (10.0%). In these participants, the highest severity rating was taken.

**DNA methylation sequences**

Genomic location (adapted from UCSC NCBI37/h19 Genome Browser) and amplified sequences of the *FKBP5* and *NR3C1* regions included in this study. CpG probes included in this study are highlighted in yellow and numbered, primer sequences are highlighted in grey. Primer sequences, including standard MassCLEAVE tags, are shown in Table 1.

**Table S1: Primer sequences**

| Amplicon | Length | Primer sequence (tags in lower case) | |
| --- | --- | --- | --- |
| *FKBP5* | 479bp | F: | aggaagagagTTGATTTAGTAGTTGGGTAAGTGGG |
|  |  | R: | cagtaatacgactcactatagggagaaggctACCCTATATCCCTCTTTTCTCCTAAA |
| *NR3C1* | 399bp | F: | aggaagagagATTTTTTAGGAAAAAGGGTGG |
|  |  | R: | cagtaatacgactcactatagggagaaggctCCCTAAAACCTCCCCAAAAAAC |

**Figure S1 - *FKBP5* sequence and probes**

Genomic location: chr6:35,695,823-35,696,542 (UCSC NCBI37/h19)


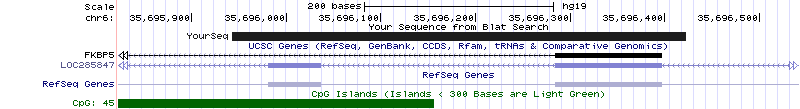


Amplicon sequence and probes

CpG 1 CpG 2

CCTGACTCAGCAGCTGGGTAAGTGGGTGTGCTCGCTCACCAGATCAACCGCTCTTGCAGCCTGC

CpG 3

GGTCAGCGACGACCCGGCACTCGGCAGCTCCCTGGAACGGGAGGGGCAAGCATACGGCCCGC

CpG 4

TCCTCTGCTCGCGCCCCAGCCTGCCGCGCGAGACCTCCACGTGGCCGGTTTGCGCATCCCTCT

GCCTTCTCGCCCCTCTATTAAAGCGTCTCCCAGCCCCCAGCCTGAGCGCAGCAGGCTCTGCCCA

CCTGGGAGAAGCACTCTCCTCACCCCACCCGACAGGTGTGGCCAGGCACACAGCTGCTGTGTA

CATGTCCTGTTTTCCCCAAATCTCACCCCTTTGGGACGCCTCGGAAGGTCTTGGCATGGGGGAT

GTAACTCTTAGTTGCAAACAGAGGTAGGAGTTTGTTCTGGGGTAGTGGTGGAGTAGGGAGCCGA

GGAAGGGGATCCTAGGAGAAAAGAGGGACATAGGGC

The FKBP5 region assayed was chosen for its proximity to the promoter region, coverage of exon 1, and overlap with nearby CpG islands (as depicted above). This amplicon also shows some overlap with the region detailed by Yehuda et al. 2013[^17^](#_ENREF_17).

**Figure S2 - *NR3C1* sequence and probes**

Genomic location: chr5:142,783,507-142,783,905 (UCSC NCBI37/h19)


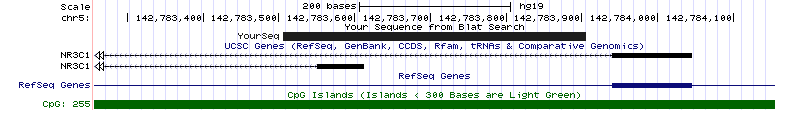


Amplicon sequence and probes

CpG1 CpG2

ACTCCCCAGGAAAAAGGGTGGCGGCGGCAGCGGCGGGGGCCGACCTGGTCTCTCTGGGGCGG

CGTTAAGAGGGCCACCGAGTTTCTCCAGTTTCTTTTCTCGCTACCTCCTTCCCGCCCCCGCCCA

GCTCCGCGGCTCCAGACCCACTCGGGAGCTCGCTCTGCCCCTTGGCGGCAAGCGCCGCCAGT

GCCCCTGCGGGTGACAGCGGGCGGGCCACAAGAGCCGGGGCGCCTCCCGCGGCTGAGCTGC

CpG 3 CpG 4

GTGAGTGGCCCGCGCCGCCGCCGCCGGGCCGAGTTGCGTGAAGTGTGTCACTTCGAAAGGGG

CTACGGGGTTGCACGGAAACGGTGCCGCAGCGTCTCGGCCGCGCTCGGGGCCGGGCGGCCTG

ACACGCCCTCTGGGGAGGCTTCAGGG

The region of the NR3C1 (GR) gene assayed was chosen for its proximity to the promoter region and nearby CpG islands. The amplicon also shows overlap with previous studies examining the effect of environmental influences on DNA methylation ^[18-20](#_ENREF_18)^. Specifically, the 6 CpG sites covered by CpG1 and CpG2 are also assayed in previous studies. The NGFI-A binding site as examined by Weaver et al [^21^](#_ENREF_21) is underlined, but CpG probes in this region did not pass our stringent quality control procedures and hence were not included in our final analyses.

**Table S2. No effect of self-reported ethnicity on genotypic proportions**

| **Gene** | **Polymorphism** | **Χ^2^** | **p** |
| --- | --- | --- | --- |
| **FKBP5** | *rs1360780* | 5.10 | 0.403 |
|  | *rs3800373* | 5.61 | 0.346 |
|  | *rs4713916* | 8.16 | 0.148 |
|  | *rs9296158* | 3.91 | 0.562 |
|  | *rs9470080* | 3.82 | 0.576 |
| **GR** | *rs6195* | 6.51 | 0.259 |
|  | *rs41423247* | 2.09 | 0.837 |

**Table S3** – Testing the effect of time-point on DNA methylation.

| Gene | CpG Site | t | df | *p* |
| --- | --- | --- | --- | --- |
| *FKBP5* | 1 | -.42 | 97 | .678 |
|  | 2 | -.05 | 97 | .299 |
|  | 3 | .46 | 92 | .644 |
|  | 4 | .74 | 96 | .461 |
| *GR* | 1 | 2.00 | 97 | .048 |
|  | 2 | .84 | 97 | .340 |
|  | 3 | .83 | 97 | .411 |
|  | 4 | .35 | 97 | .731 |

Note. No significant statistical differences between pre-treatment and post-treatment percentage DNA methylation were detected at any CpG site. Tested using paired sample t-tests.

**Table S4** – Testing the effect of pre-treatment percentage DNA methylation on change in primary anxiety severity.

| Gene | CpG Site | β | CI | *p* |
| --- | --- | --- | --- | --- |
| *FKBP5* | 1 | -0.05 | -0.14-0.04 | 0.269 |
|  | 2 | 0.01 | -0.04-0.06 | 0.647 |
|  | 3 | 0.00 | -0.05-0.04 | 0.965 |
|  | 4 | -0.04 | -0.07-0.00 | 0.054 |
| *GR* | 1 | 0.1 | -0.02-0.21 | 0.091 |
|  | 2 | 0.00 | -0.03-0.04 | 0.847 |
|  | 3 | 0.05 | -0.02-0.13 | 0.178 |
|  | 4 | 0.00 | -0.01-0.02 | 0.462 |

Note. Analyses were conducted using linear mixed effect models, including age (centred), sex and pre-treatment anxiety severity (centred) as covariates, and treatment trial as a higher order random effect.

**Interaction analyses**

**Table S5** – Testing the effect of genotype on DNA methylation.

| Gene | CpG Site |  | t | df | *p* |
| --- | --- | --- | --- | --- | --- |
| *FKBP5* | 1 | Pre-treatment | -.82 | 94 | .413 |
|  |  | Change | 1.51 | 91 | .134 |
|  | 2 | Pre-treatment | .18 | 94 | .855 |
|  |  | Change | .80 | 91 | .425 |
|  | 3 | Pre-treatment | .27 | 92 | .785 |
|  |  | Change | .57 | 86 | .568 |
|  | 4 | Pre-treatment | 1.43 | 94 | .157 |
|  |  | Change | -.73 | 90 | .468 |
| *GR* | 1 | Pre-treatment | -.05 | 90 | .962 |
|  |  | Change | -1.01 | 88 | .313 |
|  | 2 | Pre-treatment | -.11 | 90 | .910 |
|  |  | Change | -.82 | 88 | .415 |
|  | 3 | Pre-treatment | .24 | 90 | .813 |
|  |  | Change | .24 | 88 | .811 |
|  | 4 | Pre-treatment | 1.16 | 90 | .250 |
|  |  | Change | -1.03 | 88 | .308 |

Note. No significant effect of genotype was detected for either pre-treatment percentage DNA methylation or change in percentage DNA methylation at any CpG site. Tested using independent samples t-tests.

**Table S6** – Testing the effect of an interaction between genotype and pre-treatment percentage DNA methylation on change in primary anxiety severity.

| Gene | CpG Site | β | CI | *p* |
| --- | --- | --- | --- | --- |
| *FKBP5* | 1 | -0.14 | -0.36-0.08 | 0.225 |
|  | 2 | -0.01 | -0.11-0.10 | 0.893 |
|  | 3 | -0.02 | -0.11-0.08 | 0.747 |
|  | 4 | -0.05 | -0.13-0.02 | 0.161 |
| *GR* | 1 | -0.06 | -0.29-0.16 | 0.573 |
|  | 2 | 0.02 | -0.05-0.09 | 0.580 |
|  | 3 | -0.03 | -0.18-0.13 | 0.725 |
|  | 4 | -0.02 | -0.04-0.01 | 0.261 |

Note. Analyses were conducted using linear mixed effect models including an interaction term, with age (centred), sex and pre-treatment anxiety severity (centred) as covariates, and treatment trial as a higher order random effect.

*Interaction between FKBP5 genotype and change in FKBP5 CpG 4 percentage DNA methylation on remission*

Primary anxiety remitters with the risk allele genotype showed a small decrease in percentage DNA methylation during treatment (change from pre- to post-treatment: -1.6%), whilst non-remitters showed an increase (change from pre- to post-treatment: +3.7%: β= .16, *p=* .019, Figure S3, tested using logistic mixed effects models with covariates and random effects as previously described). No difference between remitters and non-remitters was detected in participants homozygous for the non-risk genotype.

**Figure S3.** Change in percentage DNA methylation at *FKBP5* CpG 4 from pre-treatment to post-treatment, by *FBKP5* genotype and remission.

1+ risk alleles

No risk alleles

1+ risk alleles

No risk alleles

**All Anxiety Remission**

**Primary Anxiety Remission**

Note. There was a nominally significant difference in DNA methylation change between primary remitters and non-remitters in participants with 1+ risk allele (β= .16, *p=* .019)

**Table S7** – Testing the effect of an interaction between change in *GR* methylation and *GR* genotype on treatment outcome.

| Gene | CpG Site | β | CI | *p* |
| --- | --- | --- | --- | --- |
| *GR* | 1 | 0.02 | -0.17-0.21 | 0.821 |
|  | 2 | 0.01 | -0.06-0.07 | 0.797 |
|  | 3 | 0.07 | -0.04-0.17 | 0.203 |
|  | 4 | 0.00 | -0.02-0.02 | 0.960 |

Note. Analyses were conducted using linear regression models including an interaction term, with age(centred), sex and pre-treatment anxiety severity(centred) as covariates, and treatment trial as a higher order random effect.

**References**

1. Rapee R, Lyneham H, Schniering C et al. . The Cool Kids® Child and Adolescent Anxiety Program. Sydney: Centre for Emotional Health, Macquarie University. 2006.

2. Rapee RM, Abbott MJ, Lyneham HJ. Bibliotherapy for children with anxiety disorders using written materials for parents: A randomized controlled trial. J Consult Clin Psychol 2006;74(3):436-444.

3. Lyneham HJ, Rapee RM. Evaluation of therapist-supported parent-implemented CBT for anxiety disorders in rural children. Behav Res Ther 2006;44(9):1287-1300.

4. Hudson JL, Newall C, Rapee RM et al. . The Impact of Brief Parental Anxiety Management on Child Anxiety Treatment Outcomes: A Controlled Trial. J Clin Child Adolesc Psychol 2013:1-11.

5. Thirlwall K, Cooper PJ, Karalus J et al. . Treatment of child anxiety disorders via guided parent-delivered cognitive-behavioural therapy: randomised controlled trial. British Journal of Psychiatry 2013;203(6):436-444.

6. Vassilopoulos SP, Banerjee R, Prantzalou C. Experimental modification of interpretation bias in socially anxious children: Changes in interpretation, anticipated interpersonal anxiety, and social anxiety symptoms. Behaviour Research and Therapy 2009;47(12):1085-1089.

7. Creswell C, Hentges F, Parkinson M et al. . Feasibility of guided cognitive behaviour therapy (CBT) self-help for childhood anxiety disorders in primary care. Mental health in family medicine 2010;7(1):49-57.

8. Spence SH, Donovan CL, March S et al. . A randomized controlled trial of online versus clinic-based CBT for adolescent anxiety. J Consult Clin Psychol 2011;79(5):629.

9. Rapee RM, Lyneham HJ, Schniering CA et al. . Cool Kids "Chilled" Adolescent Anxiety Program. Sydney: MUARU, Macquarie University. 2006.

10. Barrett PM. FRIENDS for Life program - Group leader's workbook for children. Brisbane, Queensland: Australian Academic Press. 2004.

11. Barrett PM, Farrell LJ, Ollendick TH et al. . Long-Term Outcomes of an Australian Universal Prevention Trial of Anxiety and Depression Symptoms in Children and Youth: An Evaluation of the Friends Program. J Clin Child Adolesc Psychol 2006;35(3):403-411.

12. Wergeland GJH, Fjermestad KW, Marin CE et al. . An effectiveness study of individual vs. group cognitive behavioral therapy for anxiety disorders in youth. Behav Res Ther 2014;57(0):1-12.

13. Kendall PC. Treating anxiety disorders in children: Results of a randomized clinical trial. J Consult Clin Psychol 1994;62(1):100-110.

14. Schneider S, Blatter-Meunier J, Herren C et al. . The efficacy of a family-based cognitive-behavioral treatment for separation anxiety disorder in children aged 8–13: A randomized comparison with a general anxiety program. Journal of Consulting and Clinical Psychology 2013;81(5):932-940.

15. Schneider S, Lavallee K. Separation Anxiety Disorder. In: C.A. E, T. O, editors. The Wiley-Blackwell Handbook of The Treatment of Childhood and Adolescent Anxiety: Wiley-Blackwell; 2013, p 301-334.

16. Nauta MH, Scholing A. Cognitieve gedragstherapie bij kinderen en jongeren met angststoornissen: een protocol van 12 sessies. Handleiding voor de therapeut. Groningen: Rijksuniversiteit Groningen (Klinische en Ontwikkelingspsychologie). 1998.

17. Yehuda R, Daskalakis NP, Desarnaud F et al. . Epigenetic Biomarkers as Predictors and Correlates of Symptom Improvement Following Psychotherapy in Combat Veterans with PTSD. Frontiers in Psychiatry 2013;4.

18. Oberlander TF, Weinberg J, Papsdorf M et al. . Prenatal exposure to maternal depression, neonatal methylation of human glucocorticoid receptor gene (NR3C1) and infant cortisol stress responses. Epigenetics 2008;3(2):97-106.

19. Perroud N, Paoloni-Giacobino A, Prada P et al. . Increased methylation of glucocorticoid receptor gene (NR3C1) in adults with a history of childhood maltreatment: a link with the severity and type of trauma. Transl Psychiatry 2011;1:e59.

20. Tyrka AR, Price LH, Marsit C et al. . Childhood Adversity and Epigenetic Modulation of the Leukocyte Glucocorticoid Receptor: Preliminary Findings in Healthy Adults. PLoS One 2012;7(1):e30148.

21. Weaver IC, Cervoni N, Champagne FA et al. . Epigenetic programming by maternal behavior. Nat Neurosci 2004;7(8):847-854.
